# Slow Cook-Off Experiment and Numerical Simulation of Spherical NQ-Based Melt-Cast Explosive

**DOI:** 10.3390/ma15072438

**Published:** 2022-03-25

**Authors:** Yongshen Li, Xue Zhao, Jiuhou Rui, Sen Xu, Shengquan Chang, Lizhe Zhai, Siqi Qiu, Yuanyuan Li

**Affiliations:** 1 State Key Laboratory of Explosion Science and Technology, Beijing Institute of Technology, Beijing 100081, China; 3120190205@bit.edu.cn (Y.L.); ruijiuhou@bit.edu.cn (J.R.); 3220190166@bit.edu.cn (L.Z.); 3220200108@bit.edu.cn (S.Q.); lyy1227678275@163.com (Y.L.); 2 School of Chemistry and Chemical Engineering, Nanjing University of Science and Technology, Nanjing 210094, China; xusen@njust.edu.cn; 3 Qingyang Chemical Industry Corporation, Liaoyang 111000, China; changshengquan@163.com

**Keywords:** ammunition engineering, spheroidization, nitroguanidine-based melt-cast explosive, slow cook-off experiment, numerical simulation

## Abstract

In order to analyze the influence of nitroguanidine (NQ) spheroidization on the corresponding characteristics of slow cook-off molten cast explosives, experiments and simulation calculations were carried out. A calculation method was established, based on a multiphase flow model to simulate the response process of spherical NQ-based molten cast explosives under slow cook-off conditions, to analyze the temperature distribution and liquid phase distribution during the reaction process, and to discuss the reaction temperature, reaction time and reaction location with the change of solid content. The study found that the slow cook-off response level of spherical NQ-based molten cast explosives is deflagration; the phase change cloud diagram can be used to determine the ignition time to obtain more accurate slow cook-off response data; when the solid content is 50%, the ignition temperature of ordinary NQ-based molten cast explosives is 454.3 K, and the ignition time is 50.0 h, while the slow-baking ignition temperature of spherical NQ-based fused-cast explosives is up to 464 K, which is an increase of 2.14%, and the ignition time is 51.8 h, which is a relative increase of 3.55%; it can be seen that the spheroidization of NQ improves the thermal safety of molten-cast explosives has a significant effect.

## 1. Introduction

With the increasing frequency of ammunition safety incidents in recent years, there were four flight deck accidents involving fires and munition explosions on US aircraft carriers between 1966 and 1988. Two hundred and twenty sailors and naval aviators were killed, seven hundred were injured, and ninety-six planes were destroyed or severely damaged [1]. Countries have begun to pay attention to how to reduce the danger of ammunition during transportation and storage, and insensitive ammunition (IM) has emerged as the times demand [2,3,4,5]. Because the main challenge of insensitive explosives is to provide high-energy materials with low detonation sensitivity and high performance at the same time, so that they have controllable, safe, and predictable behaviors [6], mixed explosives based on low-vulnerability elemental explosives have become the main development direction of insensitive explosives. Given the importance of damage power and cost, approximately 70% of warheads are filled with melt-cast explosives [7], so research into insensitive melt-cast explosives is critical.

Nitroguanidine (NQ) is a popular product due to its superior safety performance when compared to 1, 3,5-Triamino-2,4,6-Trinitrobenzene (TATB) and 1, 3,5-Trinitro-1,3,5-triazinane (RDX). For example, NQ has a friction sensitivity of 0, while TATB has a friction sensitivity of 4% and RDX has a sensitivity of up to 76% [8], while the energy of NQ is higher than that of TATB, which is close to RDX. What is more, NQ has a low price, as well as its non-hygroscopic and low toxicity, making it an important insensitive fundamental explosive [9]. Industrial grade NQ, on the other hand, is a hollow crystal form with a thin long needle-shaped appearance and a very low bulk density (0.15–0.25 g/cm^3^) [10]. The shape, size, and structure of energetic materials are well known to have a significant impact on their sensitivity, thermal decomposition, and bulk density [11]. NQ must be modified into a high-quality, high-bulk-density spherical crystal before it can be directly applied to the development of high-performance hybrid explosives. Thiel [12] used the cooling recrystallization method to prepare the spherical high bulk density NQ with a bulk density of 0.95–1.10 g/cm^3^. TNT (2,4,6-trinitrotoluene) has the flaws of high toxicity and simple oil leakage, and DNAN (2,4-dinitroanisole) has lower friction sensitivity and shock wave sensitivity than TNT. For example, the shock wave sensitivity of DNAN is 0, whereas that of TNT is 4–6% [8]. Currently, the US has prepared an insensitive mixed explosive IMX-101 with DNAN as a carrier and a solid content of NQ of 40% [13], but it failed the slow roasting test evaluation in the safety test of insensitive ammunition [14].

Zeng Jia [15] conducted a cook-off test of DNAN-based melt-cast explosives at three different heating rates of 0.055, 1.0, and 2.0 K/min, as well as simulating and calculating the roasting of DNAN-based melt-cast explosives at different heating rates while taking the phase transition of DNAN into account. The results showed that the ignition time at 0.055 K/min is 28,143 s, while it is only 7332 s at 2 K/min. Deng Hai [16,17] assessed the effects of various constraints on the slow cook-off properties of molten-cast explosives. The experimental data show that, as the shell sealing improves, the reaction intensity of molten-cast explosives increases accordingly. Some researchers [18,19] investigated the effect of charge size on the ignition time and temperature of DNAN melt-cast explosives when heated at a constant rate of 1 K/min. The numerical simulation findings demonstrate that when the aspect ratio of DNAN-based melt-cast explosives grows from 0.5 to 4.0, the response temperature falls from 482 K to 353 K, and the slow baking response characteristics vary considerably with charge size.

The above studies mainly focused on the influence of external parameters such as heating rate on the combustion results of mixed explosives. However, there are few studies on the influence of crystal morphology of explosives and the thermal safety of mixed explosives. To investigate the effect of NQ spheroidization on the thermal safety of mixed explosives, as well as to assess the impact of raising the NQ solid content in spherical NQ-based melt-cast explosives on their thermal safety, this paper compares the temperature changes and response conditions of spherical NQ/DNAN melt-cast explosives and ordinary NQ/DNAN molten-cast explosives with the same solid content through slow-baking experiments and simulations, finally concluding with the influence rule of spherical NQ on the thermal safety of explosives. When the common NQ-based melt-cast explosive reaches the maximum solid content of 50% in actual use, the stability under the same heating conditions is significantly different from that of the spherical NQ-based melt-cast explosive, with a shorter ignition time of 6400 s. As a result, spherical NQ should be prioritized when designing the formulation of NQ-based molten-cast explosives with the same solid content. This paper can provide a reference for the future design of high-performance spherical NQ-based insensitive explosive formulations, and to improve damage power as much as possible while meeting insensitive explosive thermal safety requirements.

## 2. Materials and Methods

### 2.1. Materials and Instruments

**Figure 1:**
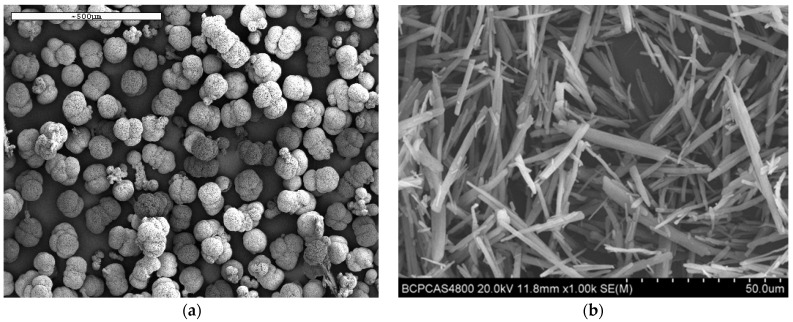
SEM of spherical NQ and ordinary NQ (**a**) spherical NQ; (**b**) ordinary NQ. Instrument: DSC1 differential scanning calorimeter, METTLER TOLEDO, temperature control accuracy is 0.1 K or less; slow baking oven, self-made; temperature controller, temperature control accuracy is 0.2 K; temperature sensor with accuracy of 0.1 K.

Material: DNAN, purity greater than 99%, Liaoning Liaoyang Special Chemical Co., Ltd (Liaoning, China). Ordinary NQ, spherical NQ (d_50_ = 500 μm) and spherical NQ with particle gradation (d_50_ = 1000, 200, 30 μm), industrial grade, Liaoning Liaoyang Special Chemical Co., Ltd. The microstructure features of the sample were characterized by a Regulus-8230 SEM (scanning electron microscope, Hitachi, Tokyo, Japan), and the results are shown in Figure 1. The slow cook-off sample column is spherical NQ-based melt-cast explosive, and the formula (mass fraction) is 75%NQ/25%DNAN. Due to its low density and poor fluidity, ordinary NQ tends to form agglomerates during melting and is difficult to charge and form. The content in melt-cast explosives is generally less than 50%. Therefore, ordinary NQ explosive columns with a solid content of 75% have not been cast this time.

### 2.2. DSC Experiment

Using a differential scanning calorimeter (TA Instruments, New Castle, DE, USA), the purge gas and the shielding gas are both high-purity nitrogen, in a stainless-steel crucible at four heating rates of 2.5, 5, 7.5, 10 K/min, in the temperature range of 373–773 K. The thermal decomposition performance of spherical NQ and ordinary NQ with a sample mass of about 1 mg is analyzed.

### 2.3. Slow Cook-Off Experiment

**Figure 2:**
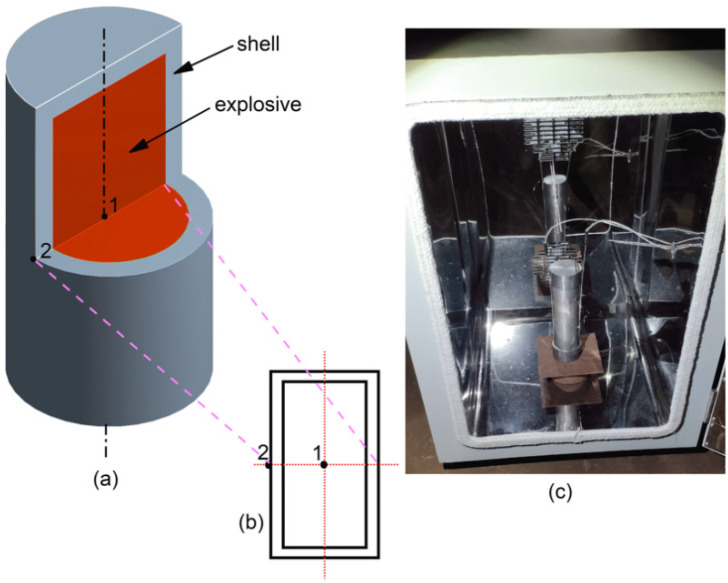
Experiment layout (**a**) Schematic diagram of the sample; (**b**) Schematic diagram of the location of the temperature measurement point; (**c**) Experimental device diagram.

In this experiment, a multi-point temperature measurement method was used to study the slow cook-off reaction of NQ/DNAN melt-cast explosive. Figure 2a is a schematic diagram of the sample. The sample is a symmetrical device, which consists of two parts: a shell and an explosive column. The shell material is 45# steel, the wall thickness is 3 mm, and the column size is ϕ20 mm × 40 mm. The sample was prepared by casting process.

Figure 2b is a schematic diagram of the location of the temperature measurement point. Two thermocouples were used to measure the temperature changes at each point of the sample during the slow cook-off process. One thermocouple was fixed at the center point of the column of the sample (measurement point 1), and the other thermocouple was fixed at the symmetry axis of the outer wall of the sample (measurement point 2).

Figure 2c is a diagram of the slow cook-off experimental device. The slow cook-off oven is responsible for the continuous heating of the sample. The temperature sensor is responsible for transmitting the temperature of the measuring point to the computer in real time, and the computer stores and generates a temperature-time curve with a temperature measurement accuracy of 0.1 K.

In this experiment, heating was started at 293 K, and the NQ/DNAN mixed explosive was heated at a constant heating rate of 3.3 K/h, until the experimental sample responded. A total of three parallel experiments were carried out to record the temperature changes at each monitoring point during the slow cook-off experiment, generate a temperature-time curve, and recover the shell and fragments after the response, based on which the response level of the slow cook-off experiment of the explosive was determined.

### 2.4. Numerical Simulation

#### 2.4.1. Mathematical Model

The transport equations of mass, momentum, and energy during the slow cook-off experiment of NQ/DNAN melt-cast explosives can all be expressed by Equation (1) [20]:

(1) $$\frac{\partial }{\partial t}(\rho \phi )+\mathrm{div}(\rho u\phi )=\mathrm{div}(\Gamma \mathrm{grad}\phi )+S$$

In the formula: *ϕ* is a general variable, representing mass, momentum, energy, etc.;* ρ* is the fluid density; *Γ* is the generalized diffusion coefficient; *t* is the time; *S* is the self-heating reaction source term of the explosive.

The self-heating reaction of NQ/DNAN melt-cast explosive follows Arrhenius’ law, and its self-heating reaction source term is the Equation (2) [21]:

*S* = *ρ*∙*Q∙A*∙exp(−*E*/*RT*) (2)

where *Q* is the reaction heat of the explosive; *A* is the pre-factor; *E* is the activation energy; *R* is the gas constant, 8.314 J/(mol∙K); *T* is the temperature.

#### 2.4.2. Physical Model

By taking the real object of the slow cook-off sample as a reference, with a symmetrical cylinder of sample, the corresponding one-half finite element model was established using ANSYS software. A hexahedral structured mesh with a mesh edge length of 0.1 mm was selected. Considering that the reaction of explosives during heating is highly complex, several assumptions were made to simplify the calculations [22]:

1. Heat conduction of the explosive only exists before melting, and there is convective heat transfer in part of the explosive column area after melting, and radiation heat transfer is always ignored;
2. The reaction kinetic parameters do not change during the heating process for the mixed explosives;
3. There is no gap between the explosive column and the shell;
4. The self-heating reactions of NQ/DNAN melt-cast explosive obey Arrhenius’ law.

The heating effect of the slow cook-off oven on the sample is simplified as the linear heating of the outer surface of the shell in the model. Heat is transferred to the inner wall of the shell through the 3 mm thick shell by means of heat conduction, and the heat transfer method of the contact surface between the inner wall of the shell and the outer wall of the explosive is defined as coupled heat transfer. The physical parameters of each component of the mixed explosive are shown in Table 1 below.

In order to study the influence of different charge formulation design and the phase change of the explosive composition on the slow cook-off process of mixed explosives, a multiphase flow model was used. Multiphase flow is a flow and heat exchange process in which a fluid and another immiscible fluid or solid are mixed with each other, where the volume fraction of phase A = volume of phase A/total volume. NQ was set as the first phase and DNAN as the second phase, and each grid unit was given the same two phase ratios, then the mass ratio of the charge design was converted to the volume ratio of the two explosives, as shown in Table 2 below. For the thermal reaction process of mixed explosives, the two phases release or absorb energy according to their own thermal decomposition reactions, and the total heat of the unit is the sum of the released and absorbed heat of each phase. If *S* is the explosive thermal reaction source term, the total heat of DNAN/NQ mixed explosive in the thermal decomposition process is *S* = *S*_DNAN_ + *S*_NQ_.

The melting point of DNAN is relatively low at 367–369 K, and its melting enthalpy is 17.87 KJ·mol^−1^ [12]. During the slow cook-off heating process, it will melt and absorb heat first, and then the auto-thermal decomposition reaction will occur. The enthalpy-porosity method that comes with the FLUENT software is used to calculate the phase score of the liquid in the DNAN melting process [15].

## 3. Results

### 3.1. DSC Experiment Results

**Figure 3:**
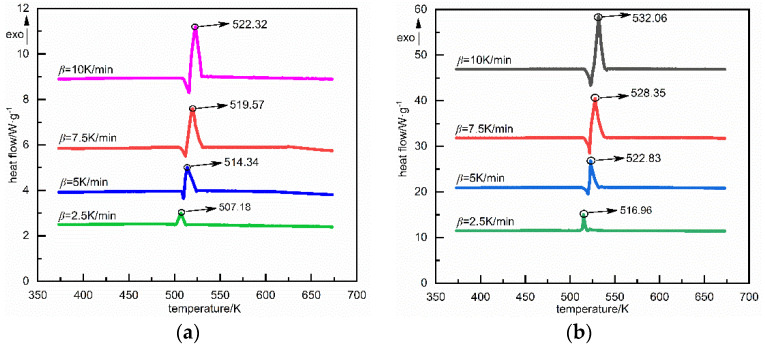
(**a**) DSC curve of ordinary NQ; (**b**) DSC curve of spherical NQ.

Figure 3 shows the DSC curves of spherical NQ and ordinary NQ at different heating rates.

As shown in Figure 3a,b, both spherical NQ and ordinary NQ have an endothermic peak at about 505 K, indicating that NQ is in a melting endothermic state near this peak, and the spheroidization of NQ does not change the melting point of explosives; at the same heating rate, the exothermic peak of spherical NQ is always larger than that of ordinary NQ, indicating that the spheroidization makes the thermal decomposition of NQ absorb more energy at a higher ambient temperature. At the same time, as the heating rate decreases, the difference between the two NQ exothermic peaks also increases significantly. This is because the spheroidization not only changes the crystal shape of the explosive, but also reduces the internal defects of the nitroguanidine crystal, and increases the probability of hot spot formation in the crystal.

**Figure 4:**
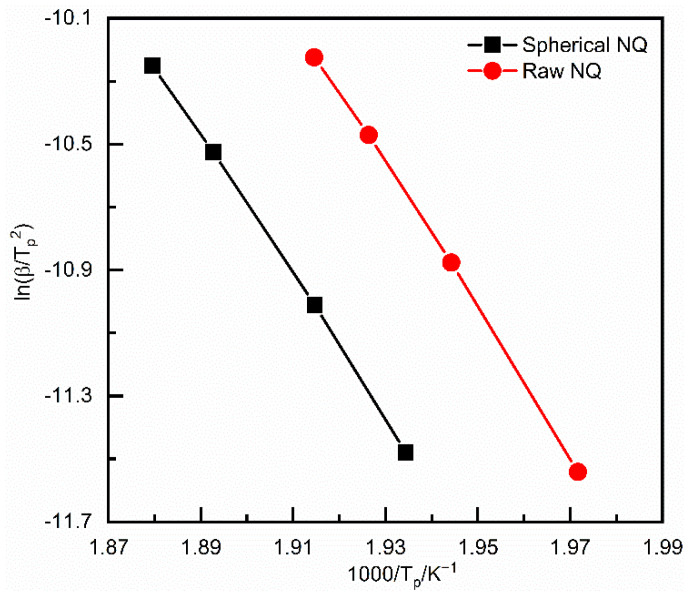
Kissinger plots for the exothermal peaks of the DSC curves of NQ.

According to the Kissinger formula [24], ln(β/T_p_) and 1/T_p_ are plotted, and the straight line shown in Figure 4 is obtained by fitting. According to the slope of the straight line, the thermal decomposition apparent activation energy Ea of ordinary NQ and spherical NQ are 158.8 kJ∙mol^−1^ and 169.3 KJ∙mol^−1^, respectively, indicating that spherical NQ requires more energy to reach the thermal decomposition reaction than ordinary NQ. It is harder to carry out, and the thermal safety is higher. The calculation results of other reaction kinetics and coefficient of correlation are shown in Table 3 below.

### 3.2. Results and Analysis of Slow Cook-Off Experiment

**Figure 5:**
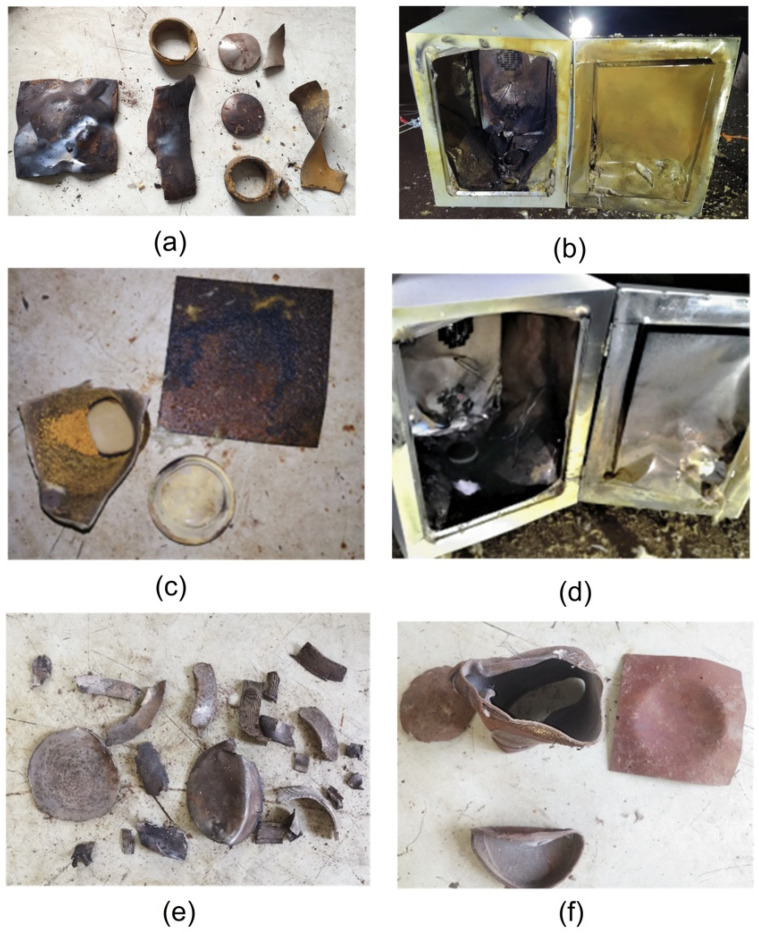
Response status of the slow cook-off experiment: (**a**,**b**) The first response status; (**c**,**d**) The second response status; (**e**,**f**) The third response status.

**Figure 6:**
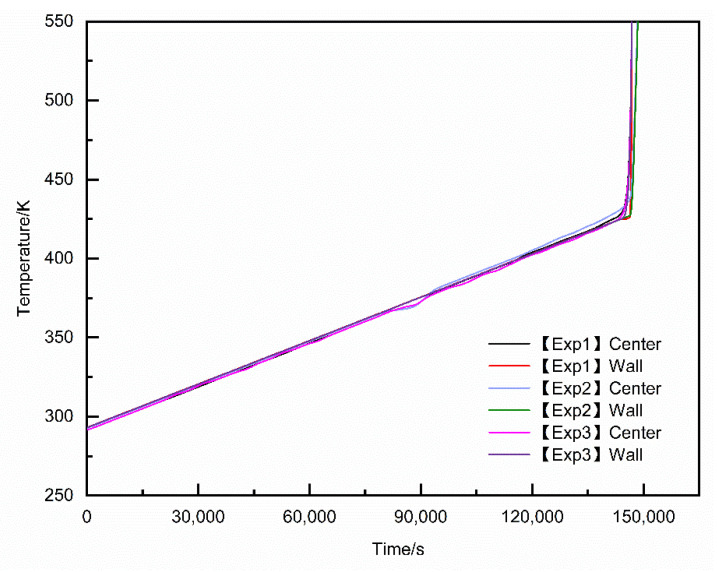
Temperature-time curve of sample experiment.

The response state of the three parallel experiments of 75% spherical NQ/25% DNAN melt-cast explosive is shown in Figure 5. From Figure 5a–f, it can be seen that the status of the device after the reaction of all slow cook-off experiments are almost the same. The slow cook-off test system deformed and showed obvious burn marks, and the upper end was torn by impact; the shell of the charge was torn apart, the charge sample was completely reacted, and the witness plate sank. However, in the first experiment, the reaction occurred when the shell temperature reached 427.3 K, while in the second experiment, the response did not occur until the shell temperature rose to 430.4 K, and the response temperature of the last set of experiment is 429.1 K. The response temperature error of the three parallel experiments is within 3 K. The temperature–time curve changes of the three experiments are shown in Figure 6. It can be seen that the temperature at the center point of the three experiments and the outer surface temperature of the shell steadily rise over time, and the temperature difference between the two temperature measurement points at the same time is not a lot, and only the temperature at the center point in the later stage of the experiment is slightly higher than the outer surface temperature of the shell, indicating that the grain has a weak auto-thermal reaction at this time. The response states of the three experiments are deflagration. The IMX-101 insensitive explosive with a formula ratio of 40%NQ/20%NTO/40%DNAN failed the slow roasting project in the previous safety assessment [13].

### 3.3. Slow Cook-Off Simulation Results

The corresponding model monitoring points are set up with reference to the temperature measurement points of the experiment, and the temperature changes at each point during the simulation process are recorded. The comparison of the experimental results and the simulation results is shown in Table 4.

It can be seen from Table 4 that the error range of each data is within 5%, which shows that the calculation model and related parameters can more accurately describe the slow cook-off experiment process of the NQ/DNAN mixed explosive.

In order to further explore the influence of NQ spheronization on the slow cook-off response characteristics of mixed explosives, three groups of spherical NQ/DNAN melt-cast explosives with different solid content (50/50, 60/40, 70/30) and ordinary NQ/DNAN melt-cast were simulated.

**Figure 7:**
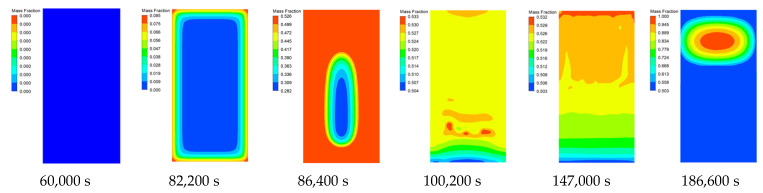
Liquid phase cloud image of 50% spherical NQ/50% DNAN at different times.

**Figure 8:**
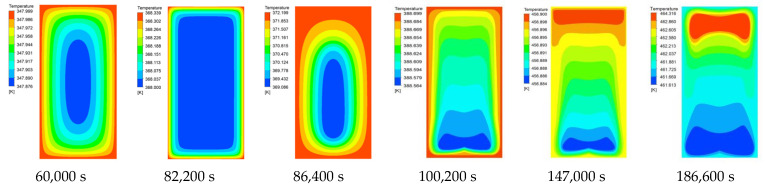
Temperature cloud diagram of 50% spherical NQ/50% DNAN at different times.

Figure 7 and Figure 8 are the liquid phase cloud diagram and temperature cloud diagram at different times during the slow cook-off simulation of 50% spherical NQ/50% DNAN melt-cast explosive, respectively.

It can be seen from Figure 7 that at 82,200 s, the temperature of part of the grains close to the shell reaches the melting point and begins endothermic melting, and the phase change occurs in the order of the explosive column surface to the inside. A total of 82,200–90,800 s is the explosive column phase change process. At this time, the density of DNAN decreases after the solid phase changes to the liquid phase. Therefore, the central part of the calculation unit that has not undergone phase change has an increase in the density of the calculation unit that has undergone phase change. Settling occurs under the action of gravity. After 100,200 s, the DNAN component in the explosive column is completely melted, but the melting point of NQ is high, so it has been maintained in a solid state. Under the combined action of gravity and the internal friction of the molten explosive column, a small part of the NQ component will settle, resulting in a liquid phase. The fraction gradually decreases in a wavy shape from the top to the bottom. The maximum liquid fraction is 0.550, which is similar to the volume fraction of DNAN in 50% NQ/50% DNAN melt-cast explosives. At 186,600s, the liquid fraction near the top of the explosive column is 1.0, indicating that the explosive column has responded. This can also be used as a basis for judging the response time and ignition position of the explosive column. Compared with the previous prediction [15], the temperature cloud graph and the sudden change point of the temperature-time curve of the ignition time and position are more intuitive and accurate.

Figure 8 shows the analysis of analyze the temperature change process of the 50%NQ/50%DNAN melt-cast explosive during the slow cook-off process. Before and at the beginning of the phase change, the temperature cloud pattern is symmetrically distributed, and the heat is evenly transferred from the surface of the explosive column to the inside. During the phase change, the temperature cloud image showed an irregular elliptical distribution with the center of gravity downward similar to that of the phase change cloud image of the same period, indicating that the unmelte central explosive column had settled. After the DNAN component was completely transformed into the liquid phase, because the unmelted NQ particles moved to the bottom, the upper explosive column produced convective heat transfer, which was much larger than the heat conduction of the lower layer, so the temperature distribution also showed a wave-like decrease from top to bottom, but heat was still transferred from the outside to the inside. At 147,000 s, the self-heating reaction occurred in the explosive column, and heat began to be transferred from the inside to the outside. With the increase of time and the accumulation of heat, the final explosive column formed an ignition center near the top, and the center temperature of the ignition position inside the explosive was 464.13 K.

Taking the liquid fraction in the phase transition cloud chart to reach 1.0 as the criterion for judging the response time of the explosive column, the simulation results of the slow cook-off of three spherical NQ-based molten cast explosives with different solid contents and ordinary NQ molten cast explosives are shown in Table 5 below.

It can be seen from Table 5 that at the same solid content of NQ, the slow cook-off simulation performance of spherical NQ-based melt-cast explosives are better than that of ordinary NQ-based melt-cast explosives. The response time of the former is increased by about 4% compared with the latter, and the growth rate even reaches 6.5% at 70% solid content. The average growth rate of response temperature is about 1.21%, which increases by 2.14% at 50% solid content. This shows that the spheroidization of NQ reduces the internal defects of the crystal and decreases the probability of the formation of hot spots during the heating process of the explosive crystal, which is beneficial to improve the thermal safety of NQ-based molten-cast explosives. The above experimental results, that the activation energy of spherical NQ is higher than that of ordinary NQ, are corroborated.

**Figure 9:**
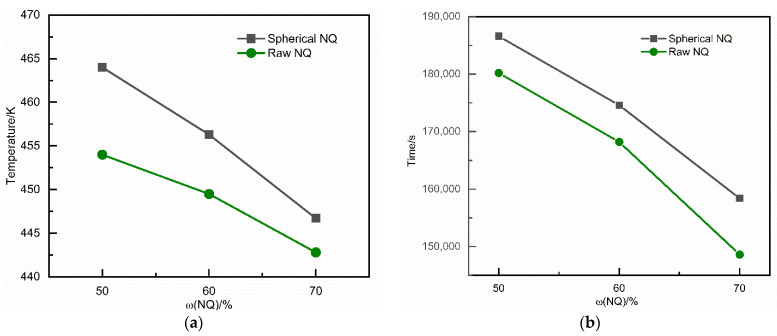
Comparison of slow cook-off results of NQ-based melt-cast explosives with different charge formulations (**a**) Ignition temperature; (**b**) Ignition time.

The following Figure 9 shows the effect of the intuitive reaction charge formula on the simulation results of slow cook-off NQ-based molten cast explosives. For spherical NQ-based melt-cast explosives with different solid content, the higher the solid content, the lower the ignition temperature and the shorter the ignition time of the slow cook-off simulation. It can be seen that the increase in solid content will reduce the thermal safety of spherical NQ-based melt-cast explosives. Ordinary NQ-based melt-cast explosives also conform to this law.

**Figure 10:**
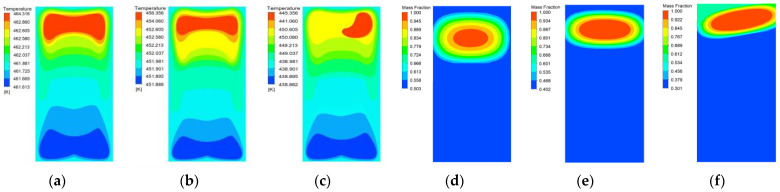
Temperature cloud diagram and liquid phase cloud diagram at the moment of ignition of spherical NQ/DNAN melt-cast explosives with different solid content: (**a**) 50%NQ/50%DNAN temperature distribution; (**b**) 60%NQ/40%DNAN temperature distribution; (**c**) 70%NQ/30%DNAN temperature distribution; (**d**) 50%NQ/50%DNAN liquid phase distribution; (**e**) 60%NQ/40%DNAN liquid phase distribution; (**f**) 70%NQ/30%DNAN liquid phase distribution.

Figure 10 shows the temperature cloud diagram and liquid phase cloud diagram at the moment of ignition of three types of solid content spherical NQ/DNAN melt-cast explosives. It can be seen from Figure 9 that the ignition position of the three solid content spherical NQ-based melt-cast explosives is close to the top of the explosive column, and with the increase of the NQ ratio, the ignition area shrinks, which shows that the convective heat transfer area is slow to ignite the melt-cast explosive. The position has a direct influence, the larger the convective heat transfer range, the larger the area of the ignition area.

**Figure 11:**
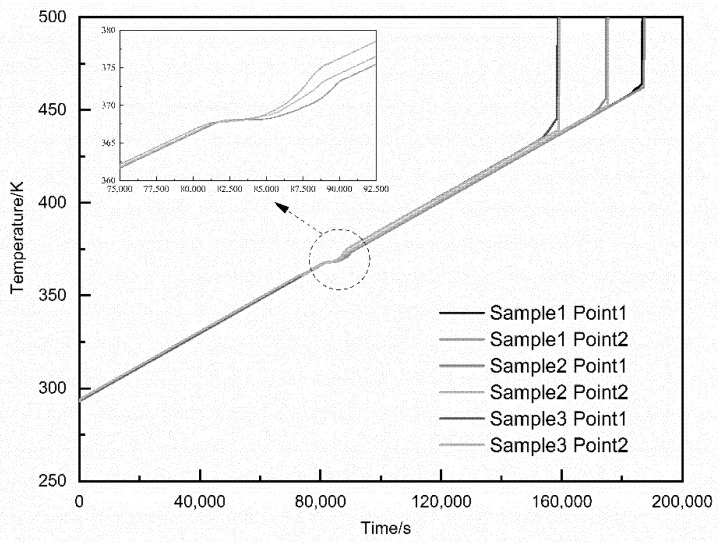
Temperature-time curve of spherical NQ/DNAN melt-cast explosive with different solid content.

Figure 11 is a temperature-time curve diagram of spherical NQ/DNAN melt-cast explosives with different solid content during the slow cook-off simulation process. From the small graph in Figure 11, it can be seen that with the decrease of DNAN content, the start time of the temperature plateau caused by DNAN melting endothermic is delayed, and the end time is advanced, so the final temperature plateau is reduced. The increase in the solid content of the sample has varying degrees of influence on the response temperature and response time of the explosive column. The response temperature ranges from 464 K to 456.3 K to 445.7 K, reduced by 1.66% and 2.32%, respectively. The response time ranges from 186,600 s to 174,600 s to 158,400 s, dropped by 6.43% and 9.28%. The increase in the solid content of spherical NQ has a more significant impact on the response time. This shows that the increase of spherical NQ content will lead to the decrease of the thermal safety of grains. Therefore, when designing insensitive ammunition formulations to increase their power, the issue of insufficient safety should be taken into consideration.

## 4. Conclusions

1. According to the experimental results, the slow-baking response degree of spherical NQ-based molten-cast explosive is deflagration; the activation energy of spherical NQ is 169.3 KJ∙mol^−1^, which is 10.5 KJ∙mol^−1^ higher than ordinary NQ, indicating that spheroidization improves the thermal safety of NQ;
2. This simulation calculation uses a multiphase flow model to more accurately describe the phase transition process in slow cook-off and to calculate the effect of NQ spheroidization on the slow cook-off response characteristics of melt-cast explosives. The starting point of the explosive response is judged by using the liquid phase score of 1.0 in the liquid phase cloud chart, which is more intuitive and has higher accuracy, and is consistent with the ignition temperature data obtained from the slow cook-off experiment;
3. It can be seen from the simulation results that when the solid content is the same, the ignition temperature of spherical NQ-based melt-cast explosives is generally higher than that of ordinary NQ-based melt-cast explosives, and the ignition time is also longer. When the solid content is the same 50%, the ignition temperature of ordinary NQ-based molten-cast explosive at response time is 454.3 K and the ignition time is 50.0 h, while the ignition temperature of spherical NQ-based molten-cast explosive is only 464.0 K, increased by 2.14%, and the ignition time is 51.8 h, also increased by 3.55%. This shows that when ordinary NQ-based melt-cast explosives reach the maximum solid content in actual use, the stability under environmental heating is significantly different from that of spherical NQ-based melt-cast explosives. When subjected to the same level of thermal stimulation on the battlefield or during daily transportation and storage, the response level of spherical NQ-based molten-cast explosives is lower, reducing the likelihood of accidental combustion or even explosion of explosives, avoiding unnecessary losses, and being more in line with insensitivity ammunition requirements for safety. As a result, when designing an insensitive melt-cast explosive with NQ as an elemental explosive, spherical NQs should be prioritized;
4. Comparing the response time temperature and ignition time of three NQ/DNAN melt-cast explosives with different solid content (50/50, 60/40, 70/30), the ignition time and ignition temperature both decreased to some extent as the solid content increased, and the higher the solid content, the more obvious the downward trend, and the same for ordinary NQ/DNAN melt-cast explosives. The reduction in ignition time and temperature means that explosives respond more quickly to thermal stimuli, lessening thermal safety and increasing the likelihood of ammunition safety accidents. Thence it can be considered that the higher the NQ content, the lower of the thermal safety of NQ/DNAN melt-cast explosives.

## Figures and Tables

**Table 1:** Physical parameters of the materials used in the calculations [23].

Materials	Density ρ/(kg∙m^−3^)	Heat Capacity Cp/(J∙kg^−1^∙K^−1^)	Conductivity λ/(W∙m^−1^∙K^−1^)	Viscosity /(kg∙m^−1^∙s^−1^)
DNAN (l)	1330	1170	0.17	0.0105
DNAN (s)	1450	1170	0.25	—
Raw NQ	1710	1222	0.442	—
Spherical NQ	1770	1222	0.442	—
Steel	7850	480	43	—

**Table 2:** Multiphase proportion of the materials used in the calculations.

Sample	Mass Percentage ω/%			Volume Percentage φ/%		
	DNAN	Raw NQ	Spherical NQ	DNAN	Raw NQ	Spherical NQ
1	50	—	50	0.549	—	0.451
2	40	—	60	0.448	—	0.552
3	30	—	70	0.343	—	0.657
4	50	50	—	0.541	0.459	—
5	40	60	—	0.440	0.560	—
6	30	70	—	0.336	0.664	—

**Table 3:** Thermal reaction kinetic parameters for the materials.

Materials	Activation Energy Ea/(KJ∙mol^−1^)	Coefficient of Correlation r	Pre-Exponential Factor *A*/(s^−1^)	Reaction Heat *Q*/(KJ∙kg^−1^)
DNAN [15]	172	—	1.2 × 10^11^	4920
Raw NQ	158.8	0.9997	6.135 × 10^11^	8500
Spherical NQ	169.3	0.9994	5.214 × 10^12^	8700

**Table 4:** Comparison of experimental results and simulation results.

	T/K		Ignition Time/s
	Point 1	Point 2	
Test	428.90	428.20	146,500
Simulation	427.89	426.14	142,400
Error (%)	0.23	0.48	2.88

**Table 5:** Slow cook-off simulation results of NQ-based melt-cast explosives with different charge formulations.

Solid Content	Spherical NQ		Raw NQ *	
	Te/K	Ignition Time/s	Te/K	Ignition Time/s
50	464.0	186,600	454.3	180,200
60	456.3	174,600	449.5	168,200
70	445.7	158,400	442.8	148,600

* Note: Ordinary NQ is easy to form agglomerates during melting and casting due to its poor fluidity, so it is difficult to charge and form, and its content in melt-cast explosives is generally less than 50%. In this simulation, in order to compare the slow cook-off response performance of spherical NQ and ordinary NQ, the solid content of 50%, 60%, and 70% was set, actual production of which cannot be realized.

## Data Availability

Not applicable.
